# Supplementary material for: Impact of High Light Intensity and Low Temperature on the Growth and Phenylpropanoid Profile of Azolla filiculoides
Source: Int J Mol Sci. 2023 May 10;24(10):8554. doi: 10.3390/ijms24108554 (PMC10218715; doi:10.3390/ijms24108554)
Supplement: Supplementary file 1 [file ijms-24-08554-s001.zip › ijms-2322564-supplementary.pdf]

Supplementary material

Tables S1. List of the genes investigated by qRT-PCR analysis and sequence of the primers employed.

Genes			Primers		
**Name**	**ID**	**Reference**	**Forward (5'-3')**	**Reverse (5'-3')**	**Reference**
Efact 1-α	AzfiRT00807	(1)	ACAGACCCAACAGAATCGGC	CTTCAACCCTTCCGCACCT	(1)
DFR1-1	*Azfi_s0035.g025620*	(2,3)	GCAGTTGGCTAGTCAAACGAC	CCCAGGGAGTTCTCTTAGAAGTG	This study
DFR1-2	*Azfi_s0245.g059984*	(2,3)	GTGAGGAACCCAGATGACCC	TCAAGCAATTGGCCTTTCACG	
DFR2-1	*Azfi_s0008.g011655*	(3,this study)	AGAGCCAGTGGGACATCGTTTTGA	CTACAACACTTGATGGCACCATCGTAC	This study
DFR2-2	*Azfi_s0008.g011657*	(3,this study)	GCTACCATTCCTCCAACGGTTATCGTAT	GCATATGTGGCGACCCTTTGCTG	
LAR	*Azfi_s0197.g057377*	(2)	GAGGGATCCTTGGATGACCATG	TGACACATCCACCTCCTTGATG	(4)
***MYB*s**	*Azfi_s0001.g000839*	(2)	CAGCAATCACACTAGCAGTAAGACCA	CCTAGCACTCTCCCATTGAGCCA	This study
	Azfi_s0129.g048859		GACACATTGGAGATTCTGGCATTGGG	AGGAAAGTTCATTGCTCTCAGGAAAGG	
	Azfi_s0005.g009154		CCTGATCCATGTCCTCAGCCATTG	GGCAACATTGGTTTCTGTAACGAGG	
	Azfi_s0007.g010874		CTAGAGATATCTGAAGTGGGTTGTAATG	CTTCATGGACTCCTTCGAGAAGTC	
	Azfi_s0016.g014344		CAGTTGCTGTAGCTCACCGTTGAA	GAACAAAGTGTGGACGTAGGCGAG	
	Azfi_s0113.g045874		AATCCATCTCTATGCGTTACACGAG	CGCCATAGCTTGATTAGTATGCCC	
bHLHs	*Azfi_s0018.g014776*	This study	TCATGCCAGAAGATCTCGCCAC	CTGTCAAATCTTGTGGAGAAGACGG	This study
	Azfi_s0004.g008875		AACTCAGGAGACAACGCGACATAG	CCCATTGAGACATTGAGACTAGATCC	
	Azfi_s0114.g046110		GAGATGGACGTGACAGACACA	AAGGAATACATCATGGAGACGAGG	
	Azfi_s0034.g025419		TAATCTCCAACACAAGCTCCAGG	CAGCTCAGAGGGCCATGTT	
	Azfi_s0277.g061489		TTCAGAAATCGGTCACGGAGG	GTGCCATTTGGGCTAATTCGG	
	Azfi_s0075.g037659		GCGTGGTCGAGCTTGGAT	GTTGAGAAATGCATGATCCTCATCG	
TTG1-1	Azfi_s4235.g118410	This study	TGATCCTGGTGATAAGACAAGATCC	GCATCCTGGTCATTGGCCATT	This study
TTG1-2	Azfi_s0016.g014399		ACAAGGTCCAGCTGAAAAGTATAATGAAC	GCTGTCATCACTGTCACTCACAA	

(1) de Vries et al. (2016)
(2)= Gungor et. (2021)

(3)= Piatkowski et al. (2020)
(4)= Costarelli et al. (2021)

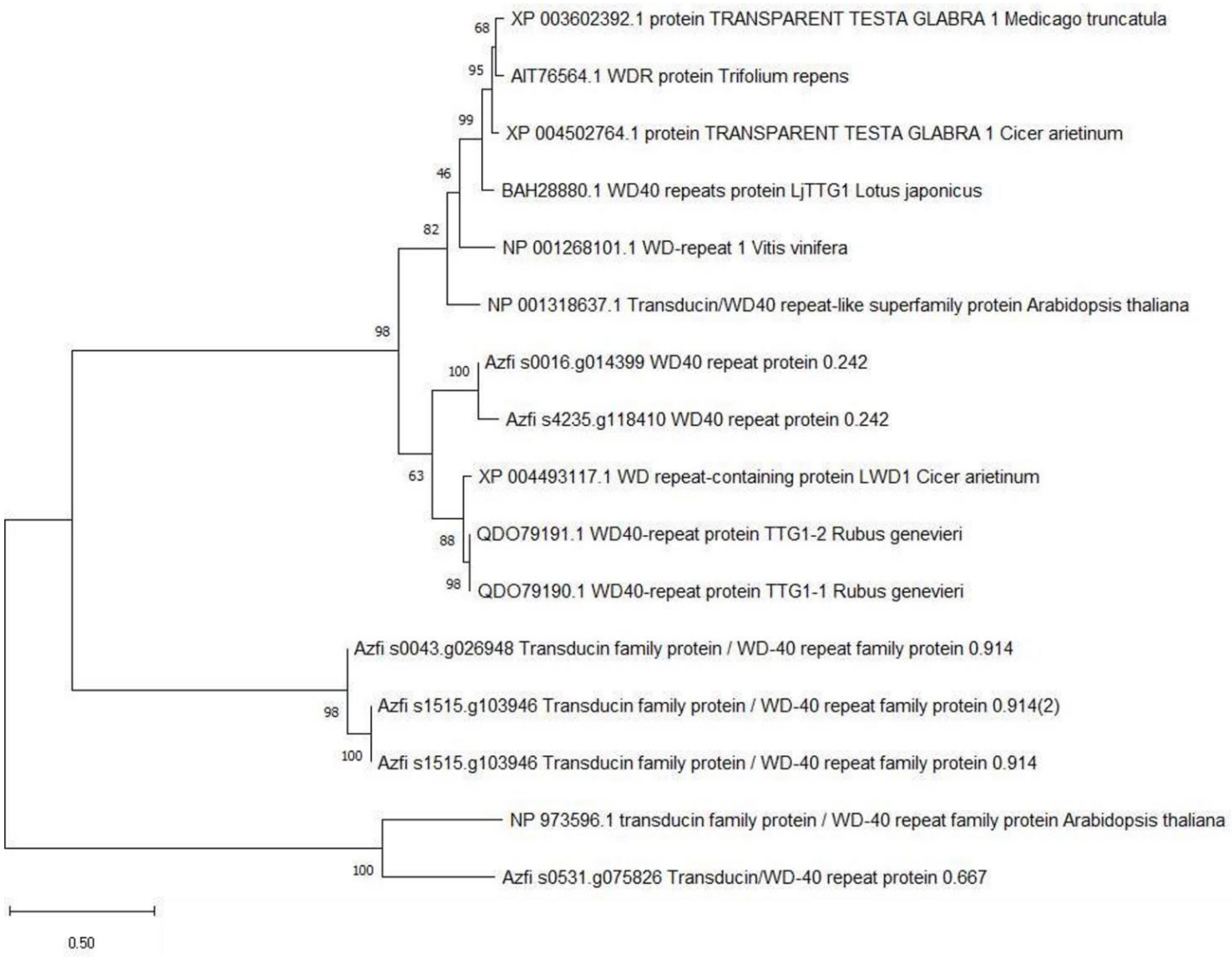

Figure S1. The phylogenetic tree of WDR proteins. The evolutionary history was inferred by using the Maximum Likelihood method and the number of the bootstrap replicated 1 000. The tree with the highest log likelihood is shown.

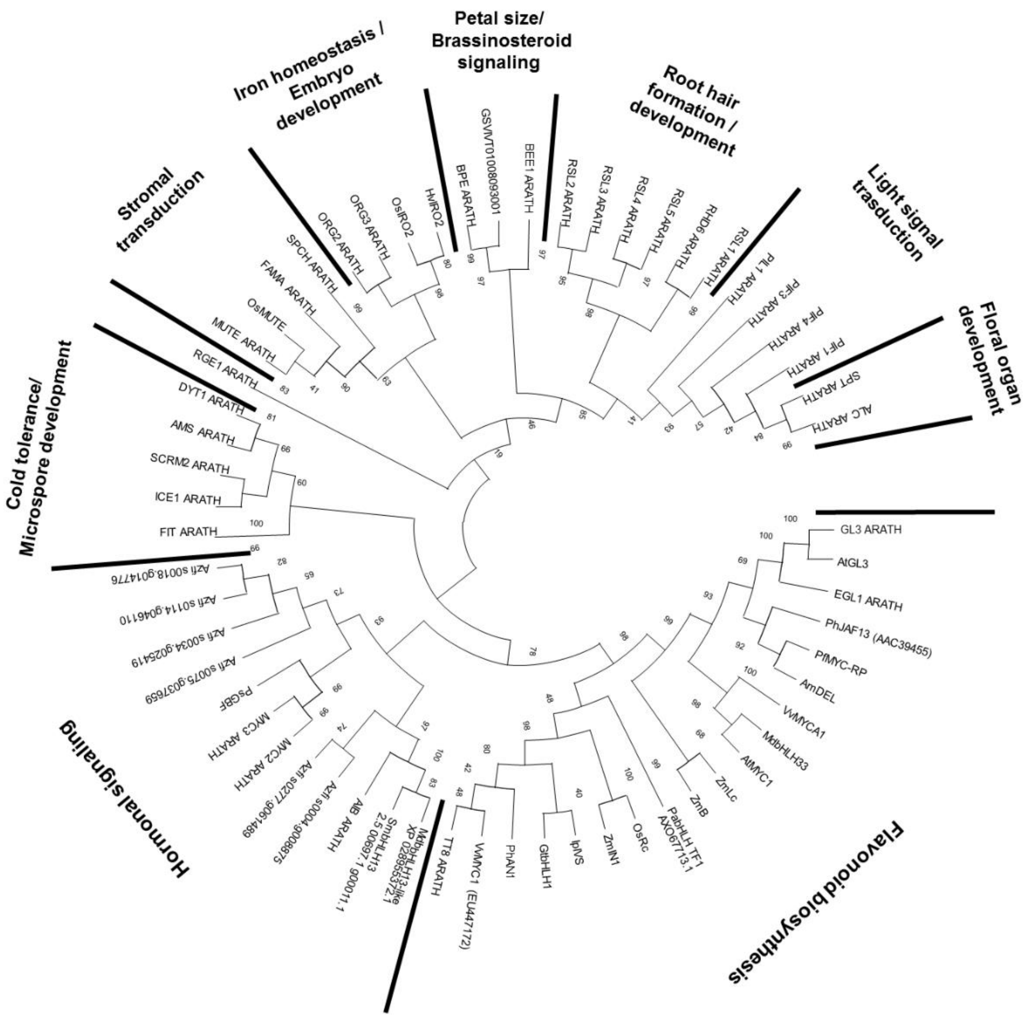

Figure S2. The phylogenetic tree of bHLH proteins. The evolutionary history was inferred by using the Maximum Likelihood method and the number of the bootstrap replicated 1 000. The tree with the highest log likelihood is shown.

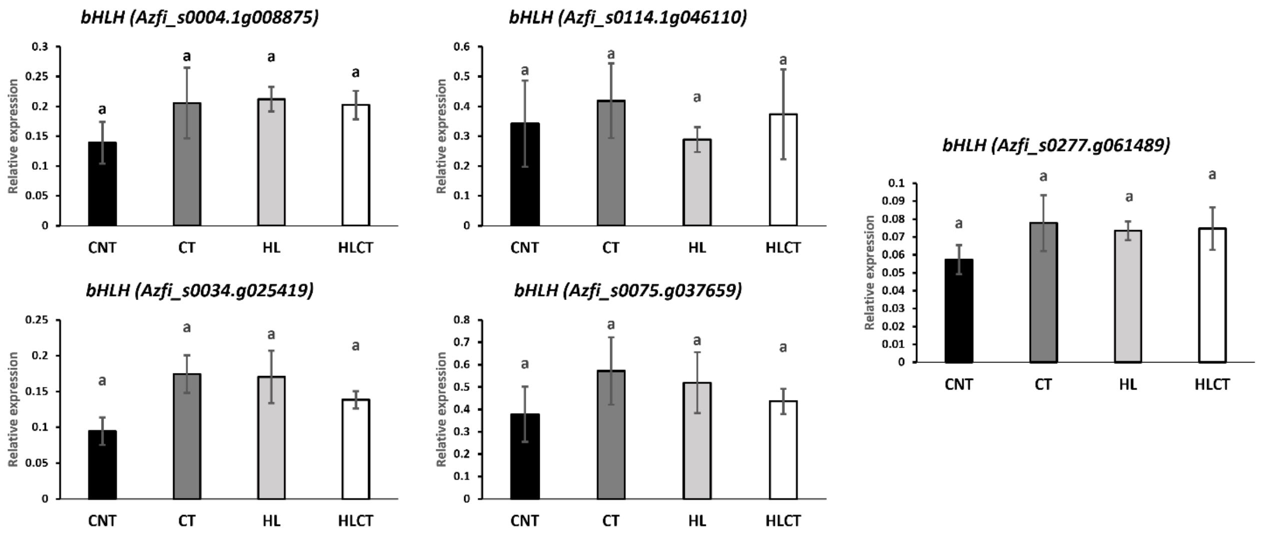

Figure S3. Relative expression levels of additional *A. filiculoides bHLH* genes assayed in this study. The relative expression of each gene is calculated using the $(2^{-\Delta Ct})$ algorithm. Significant differences determined by ANOVA followed by Tukey's multiple comparison test ($P < 0.05$ for both) are indicated by different letters.

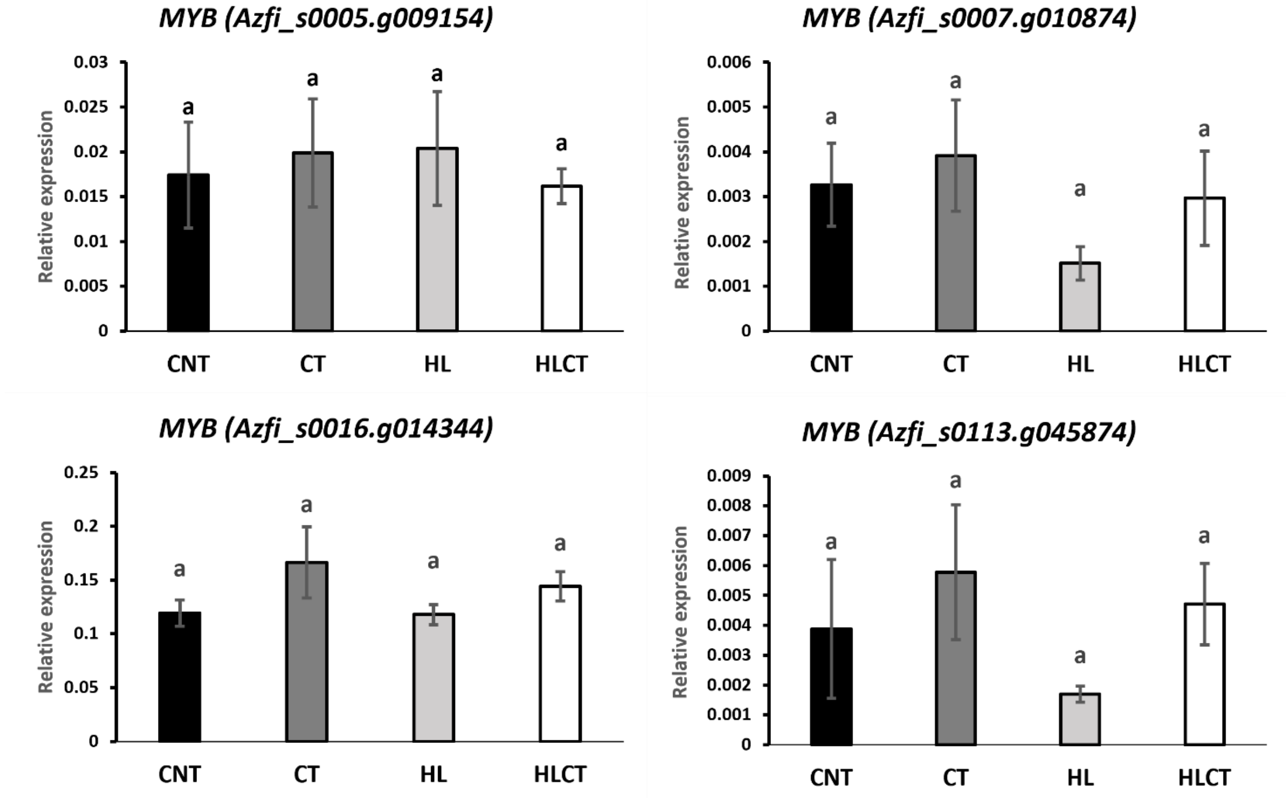

Figure S4. Relative expression levels of additional *A. filiculoides MYB* genes assayed in this study. The relative expression of each gene is calculated using the $(2^{-\Delta Ct})$ algorithm. Significant differences determined by ANOVA followed by Tukey's multiple comparison test ($P < 0.05$ for both) are indicated by different letters.
